# Cancer drugs in LMICs: cheap but unaffordable

**DOI:** 10.18632/oncotarget.21976

**Published:** 2017-10-24

**Authors:** Bishal Gyawali

**Affiliations:** Institute of Cancer Policy, London and Department of Clinical Oncology and Chemotherapy, Nagoya University Hospital, Nagoya, Japan

**Keywords:** value, affordability, global oncology, drug prices, cancer

The high cost of drugs and the therapeutic value they offer have been a matter of intense debate and scrutiny in recent times. Value is the benefit received for the cost paid. In recent years, ASCO and ESMO have developed value assessment tools (the ASCO value framework [1] and the ESMO Magnitude of Clinical Benefit Scale [2]) to guide assessment of benefit from a given cancer drug tested in trials. Although these scales are helpful to compare clinical benefits with cancer drugs, how much the health care system is ready to pay for a given benefit is a difficult question that each health care system must decide for itself. However, intricate to value, are the questions of affordability and accessibility. Affordability refers to whether or not the buyer has the financial means to purchase a particular drug, irrespective of the value. There can be high value cancer drugs such as imatinib for chronic myeloid leukemia which can still remain unaffordable due to its high cost. Finally, a drug could be both high value and affordable, but inaccessible because the drug is unavailable due to regulatory restrictions, or decreased production because the industry lacks the financial incentives to keep manufacturing cheaper chemotherapeutic drugs.

The article by Godlstein et al. published in the October issue of this journal is an important work that looks at the affordability variable [3]. In their analysis, they include a cohort of seven countries-spanning from low and middle income to high income, and from all the continents but South America. The cost of cancer drugs, as we all knew, were not similar across the countries. India has been well known in the global oncology community as the country where cancer drug prices are cheaper compared to other countries. For instance, the 4 weekly cost of trastuzumab was $2761 in India versus $6849 in the U.S [3]. However, despite trastuzumab being a high value drug for HER2 positive breast cancer and costing cheaper in India, more than 99% of patients with HER2+ breast cancer didn’t receive trastuzumab in an Indian trial [4]. This is probably because cheaper in comparison to U.S is still too expensive for most Indian patients living on an average Indian salary. Thus, cheaper drug prices don’t necessarily translate to affordability and the analysis by Godlstein et al demonstrates this clearly. They show that the monthly drug prices were the highest in the U.S and lowest in India. However, despite having the lowest drug prices, drugs were the least affordable ( affordability estimated as drug prices divided by GDP per capita or average salary) in India.

This acknowledgment of unaffordability of cancer drugs in low and middle income countries (LMICs) despite having cheaper drug prices is only the first step. Differential drug pricing based on the purchasing capacity of the population could be a reasonable solution forward. Another solution would be to differentiate the highly effective cancer drugs (what the authors call Highly Active Anti-Cancer Therapies (HAACT) [3]) from low value drugs. Once these HAACT drugs are identified, concerted efforts are essential to ensure that these HAACT drugs are made available and affordable globally. This needs changes in policy at national and international levels, including the WHO. The WHO Essential Medicines List (EML) can act as a guide to prioritize the cancer drugs that should be affordable and accessible to every patient irrespective of geographic location or financial reserve. Indeed, the addition of imatinib and trastuzumab to the WHO EML list is a big step forward. The next step is to ensure their affordability and accessibility, otherwise there is a threat of the WHO EML being relegated to “just another list”.

Although cancer drugs are our concern, the policy makers should ensure that access to minimally life extending drugs don’t come at the expense of life saving surgeries and radiotherapy services for early stage cancers or preventive services against cancer. For instance, the focus of LMICs cancer policy against cervical cancer shouldn’t be about how to ensure access to bevacizumab for advanced cervical cancer but to ensure wide coverage to HPV vaccination and screening programs to reduce the number of patients that present with advanced cervical cancer [5]. The governments and policy makers in LMICs should prioritize access to highly effective anti cancer drugs used in curative setting and limit spending on costly but ineffective or minimally effective drugs used in palliative setting [6]. Those drugs that ensure cure should be given the first priority. It was indeed heartening to see that India paid $19006 for a 4 week course of bevacizumab (based on purchasing power parity) while Australia paid only $543 [3]. There can be no justification whatsoever that India can spend so lavishly on a marginal drug like bevacizumab for some patients but unable to ensure access to trastuzumab to all patients with HER2 positive breast cancer.

In conclusion, drug prices are lower but still unaffordable for people living in LMICs. Efforts should be concerted towards ensuring global access to highly effective anticancer drugs. LMICs should prioritize the curative or potentially curative cancer drugs and not simply copy paste the high income countries where billion dollars are spent on a drug that provides marginal gains [7].

https://twitter.com/oncology_bg
